# Changes in peer and sibling victimization in early adolescence: longitudinal associations with multiple indices of mental health in a prospective birth cohort study

**DOI:** 10.1007/s00787-020-01708-z

**Published:** 2021-01-11

**Authors:** Helen Sharpe, Elian Fink, Fiona Duffy, Praveetha Patalay

**Affiliations:** 1 School of Health in Social Science, University of Edinburgh, Teviot Place, Edinburgh, EH8 9AG UK; 2 Faculty of Education, University of Cambridge, 184 Hills Road, Cambridge, CB2 3PQ UK; 3 Centre for Longitudinal Studies and MRC Unit for Lifelong Health and Ageing, University College London, Gower Street, London, UK

**Keywords:** Victimization, Depression, Life satisfaction, Body image, Self- esteem

## Abstract

Victimization by peers and siblings is associated with poorer mental health outcomes in adolescence. What is less clear is whether mental health outcomes improve if victimization experiences cease (e.g., being victimized in primary school but not secondary school). This study aims to explore how changes in victimization experiences are associated with changes in mental health outcomes in early adolescence. Data are from 13,912 participants in the Millennium Cohort Study (MCS), a nationally representative cohort of individuals born in the UK. Self-reported victimization by peers and siblings, as well as mental health outcomes (depressive symptoms, life satisfaction, self-esteem, and body image), were collected at age 11 and age 14. Victimization at either time point was associated with poorer mental health across the range of outcomes, with effects largest for those who were consistently victimized. Those who reported increasing victimization had greater deterioration in their mental health compared with their peers who were never victimized. Conversely, children whose victimization decreased showed similar mental health development over this period as those who were never victimized. There was a cumulative effect of victimization by peers and siblings, with effect sizes generally larger for experiences with peers. Victimization in adolescence is associated with enduring reductions in mental health. Nonetheless, the promising outcomes associated with reductions in victimization suggest the potential for bullying interventions in schools to limit the deterioration in mental health in victimized groups.

**Supplementary Information:**

The online version contains supplementary material available at 10.1007/s00787-020-01708-z.

## Introduction

Bullying victimization is a specific form of repeated aggressive behaviour with an intent to cause harm that occurs in the context of a power imbalance [1]. Around one in ten adolescents report experiencing frequent bullying victimization [2], and these experiences are a well-established risk factor for poor mental health, with concurrent and long-term negative consequences [3]. Importantly, the experience of victimization is not necessarily stable over time, with victimization potentially increasing or decreasing across the lifetime [4]. The transition from primary education to secondary education (which in the UK occurs around age 11 years) offers a particular period of interest for research as changing school contexts and peer groups in this time may precipitate changes in victimization experiences [5]. Although the mental health implications of being victimized are well established, less well understood are the consequences of changes in the experience of victimization for mental health (e.g., becoming a victim, or no longer being victimized). Furthermore, although the majority of research has focused on victimization in the school context, recent work has highlighted the longitudinal influence of sibling victimization for child mental health [6]. The current study investigates the mental health implications of both peer and sibling victimization across the transition from primary to secondary school, exploring how changes in these two different sources of victimization independently and in combination predict a range of different indices of adolescent mental health.

There is robust evidence of an association between peer victimization in childhood and mental health difficulties [3], with increasing longitudinal evidence suggesting a prospective link between earlier peer victimization and later mental health problems [7, 8]. For example, depression has consistently been found to be associated with victimization [9], with increasing frequency of victimization experiences associated with increased severity of depression [7]. A similar pattern of findings have been demonstrated between peer victimization and other indicators of mental well-being, such as poor self-esteem [10], body dissatisfaction [11], and poor subjective well-being [12]. Furthermore, research has found differential outcomes for those children who experience chronic peer victimization and those who experience peer victimization limited to a discrete period of development [13, 14].

While much of the research on the negative outcomes of victimization focuses on victimization by peers, in recent years, a focus on sibling conflict and victimization has come to the fore [15], particularly the implications of sibling victimization for children’s mental health both concurrently and longitudinally [6, 16]. For example, Bowes and colleagues found that those children who were bullied by their siblings at 12 years of age had a greater likelihood of depression, anxiety, and self-harm at 18 years of age [6]. There are several factors that make studying sibling victimization important. First, the frequency of this type of victimization is reported as being greater than that of peer victimization, which may reflect the fact that sibling relationships typically comprise frequent contact and an inherent power imbalance [16, 17]. Second, sibling and peer victimization do not occur independently. Over half of adolescents reporting victimization by siblings also report victimization by peers [18], and recent evidence suggests that sibling victimization results in an increased likelihood of peer bullying in middle childhood and adolescence [6, 19]. Third, children victimized both at home and school reported greater levels of mental health problems compared to those victimized in a single context only [20], suggesting an additive effect of multiple sources of victimization [17, 18, 21, 22].

While for most children home and family represents a fairly stable context for victimization, the transition from primary to secondary school is a particularly important time to understand peer victimization as young adolescents transitioning to secondary school are at a greater risk of being bullied than students in older year groups [23, 24]. Furthermore, this transitional period offers a powerful opportunity to explore the impact of changes in victimization for mental health, as research suggests that the peer victim role in this period is relatively unstable [4]. The intersection of peer and sibling bullying may also come to the fore at this time as positive family social support, particularly at the transition to secondary school, is shown to promote positive student transition experiences and well-being [25, 26] as well as resilience to bullying [27]. Those families that lack parental warmth and support are also more likely to exhibit sibling bullying [21]. Importantly, sibling relationship quality over and above parent–child relationship has a protective effect on the well-being of children victimized in a peer context, with those children experiencing peer bullying at school and sibling bullying at home experiencing a twofold blow for mental health.

Finally, in studies that examine victimization and mental health, it is crucial to include a focus on child sex, as both the nature and frequency of peer [2, 28] and sibling victimization experiences [6] have shown to differ by sex. Furthermore, differences between boys and girls are also apparent in both the type and prevalence of mental health problems [29]. Finally, there is some evidence that the mental health implications of victimization also appear to show sex differences [30]. Together, these results emphasise the importance to taking into account sex when examining the association between victimization and mental health.

In the current study—the largest to our knowledge that examines both sibling and peer victimization and the only study to examine both sources of victimization longitudinally—we aim to examine how changes in victimization across both home and school are associated with changes in a range of indices of mental health between 11 and 14 years of age. We focus on frequent victimization (at least once a week) and changes in the experiences of this to focus our understanding on the potential intervention benefits of targeting and reducing the experience of frequent bullying victimization during these formative years.

Given the strong evidence that victimization precedes poor mental health [8], we hypothesised that: (a) those who report being victimized at both time-points will report the poorest mental health outcomes, (b) those who report victimization at neither time point will report the most positive mental health outcomes, and (c) those who report the onset of being victimized between ages 11 and 14 will show deterioration in mental health outcomes over this time period. Given the lack of preceding evidence, we did not make specific hypotheses regarding the group who reported being victimized at age 11 but not at age 14. Regarding victimization across contexts, we hypothesised that: (d) the impact of victimization in different contexts will be cumulative (i.e., victimization by both peers and siblings will be associated with poorer mental health than victimization by either in isolation). We did not make specific hypotheses as to whether the interplay between peer and sibling victimization on mental health outcomes will be independent (additive) or multiplicative. Finally, given previous evidence, we hypothesised that: (e) the association between victimization and mental health outcomes will vary by sex with stronger associations in girls compared with boys.

## Method

### Study design and setting

Participants are drawn from the Millennium Cohort Study (MCS), a birth cohort of 19,517 individuals born in the UK between September 2000 and January 2002 [31]. The current study uses data from wave 5, collected between January 2012 and February 2013 (age 11), and wave 6, collected between January 2015 and March 2016 (age 14). Ethical permission for each wave of data collection was received as described in the study documentation. More information on the study design, variables, and response can be found at: http://www.cls.ioe.ac.uk.

### Participants

For the current study, all cohort members who participated in either the age 11 or age 14 survey were included. In families with twins and triplets, one child was randomly included. Participants were more likely to be missing data if the cohort member was male, of Black ethnicity, had parents from a lower occupational and educational level and from a single parent family [32]. Sample and attrition weights created for the study were used in all analyses to provide nationally representative estimates.

### Variables

All exposure and outcome variables were assessed at both ages 11 and 14. Any differences in the measurement across the two time-points are noted below.

#### Victimization

Victimization by peers was assessed using the item “How often other children hurt or pick on you on purpose” (response options: most days; about once a week; about once a month; every few months; less often; never). To be categorized as experiencing victimization, a frequency of at least “about once a week” needed to be selected. Similarly, participants were coded as being victimized by siblings if they responded to the item “How often brothers or sisters hurt or pick on you on purpose” them with a frequency of at least “about once a week” [33]. The original distribution across all response categories for each variable is shown in Supplementary Materials, Table S1.

Given the aim of this paper was to estimate the potential benefits of reductions in frequent victimization, we created two separate categorical groups for peer and sibling victimization experiences by combining respective responses from the age 11 and 14 surveys, so that each participant was categorized as experiencing either: consistently low victimization (not victimized at either age 11 or age 14), consistently high victimization (victimized at both ages 11 and 14), increasing victimization (not victimized at age 11 and victimized at age 14), and decreasing victimization (victimized at age 11 and not victimized at age 14) across both peer and sibling victimization [15, 19]. For those children without siblings, for the sibling victimization categorical variable, we included an additional ‘no siblings’ group.

#### Depressive symptoms

Depressive symptoms at age 11 were assessed by asking participants to rate on a five-point scale from ‘Never’ to ‘Almost always’, how often in the past 4 weeks they felt “sad”, “afraid or scared” and “worried about what would happen”. These three items had good internal reliability (*α* = 0.73) and the mean of these three items was used as an overall depressive symptoms score. At 14, depressive symptoms were assessed using the Short Mood and Feelings Questionnaire (SMFQ) [34]. The SMFQ is a 13-item measure which asks participants to rate how they have been feeling in the past 2 weeks, with items such as “I felt miserable or unhappy”, and “I cried a lot”. All items were rated on a three-point scale: ‘not true’, ‘sometimes’, and ‘true’ (*α* = 0.93).

#### Life satisfaction

Overall satisfaction with life was measured with the item “On a scale of 1 to 7 where ‘1′ means completely happy and ‘7′ means not at all happy, how do you feel about the following parts of your life: Your life as a whole?”.

#### Self-esteem

Self-esteem was assessed using a short version of the Rosenberg Self-Esteem Scale [35]. Participants responded to five items, such as “On the whole, I am satisfied with myself” on a four-point scale from ‘strongly disagree’ to ‘strongly agree’ (*α* = 0.90).

#### Body image

Body image was measured with the following item: “On a scale of 1 to 7 where ‘1′ means completely happy and ‘7′ means not at all happy, how do you feel about the following parts of your life: The way you look?”.

#### Control variables

Demographic characteristics included age, sex, ethnicity (White, Mixed, Indian, Pakistani/Bangladeshi, Black, other), and family income (OECD equivalized income quintiles), living in a two-parent household. In addition, physical growth was accounted for using BMI percentiles, calculated using gender and age norms using the British 1990 Growth Reference, and pubertal development, using the Pubertal Development Scale [36] as reported by the participant at age 14.

### Statistical methods

All analyses were conducted using STATA v14. We used linear regression models to examine the association between the groups of victimization experiences (consistently high, consistently low, increasing, and decreasing) and (a) absolute levels of each of the four outcomes (depressive symptoms, life satisfaction, self-esteem, and body image) at age 14, and (b) changes in each of the four outcomes between ages 11 and 14. All of the outcomes were standardized to allow for comparison of coefficients between the models. The coefficients reported therefore represent the change in standard deviations in each outcome. The two victimization variables were entered separately into models initially and then entered simultaneously to examine the independent association of each. Interactions terms (victimization by peers * victimization by siblings) were also examined to investigate potential multiplicative effects. To examine whether associations differed in males and females, we investigated interactions between victimization grouping and sex on all outcomes. All models were adjusted for age, ethnicity, family income, living in a two-parent household, being an only child, prior emotional problems (parent-reported Strengths and Difficulties Questionnaire Emotional Problems Subscale at age 8) [37], special educational needs, long-term illness, BMI percentile at age 11, BMI percentile change between age 11 and age 14, and pubertal development. Given multiple testing, alpha threshold is reported at 0.01.

Approximately 8% of the sample were only children. As the research questions related to peer and sibling victimization both independently, as well as in combination, we chose not to exclude only children from analyses (see information above on coding of sibling victimization in this group). However, as there is evidence that victimization experiences may be different for only children [e.g., 38, we conducted sensitivity analyses excluding only children (see Supplementary Materials, Tables S6 and S7)]. The conclusions of the study did not change so the full sample is reported here.

Missing data across the four outcomes ranged from 7.88 to 8.87% at age 11, and from 19.97 to 20.18% at age 14. Similarly, rates of missing data for victimization by peers and siblings were 7.91% and 8.05% at age 11, and 19.70% and 19.14% at age 14, respectively. Multiple imputations with chained equations were used to impute missing data (20 imputations, 200 iterations) for all variables with missing values (i.e., all variables apart from sex, ethnicity, living in a two-parent household, being an only child). Survey and attrition weights were used to account for the clustered, stratified sampling design and the non-random attrition in the MCS [32].

## Results

### Participants

There were 13,912 participants in the analytic sample. Of these, 49.34% were female, and the majority were of White ethnicity (80.29%), with 9.51% of Asian ethnicity, 4.50% mixed ethnicity, 3.38% Black/Black British, and 2.32% of other ethnic backgrounds. The mean age of the sample was 11.17 years (*SD* = 0.33) at the age 11 survey and 14.26 (*SD* = 0.34) years at the age 14 survey.

### Descriptive data

Descriptive statistics for the exposures and outcomes are shown in Table 1. Participants were more likely to report being victimized by siblings compared to peers. In relation to both peer and sibling victimization, the largest group was formed by children who were not victimized at either age 11 or age 14 (peers: 77%; siblings: 44%). For those children that were victimized, 12% experienced a decrease in peer victimization and 23% experienced a decrease in sibling victimization between age 11 and age 14, while 7% experienced an increase in peer and 9% experience an increase in sibling victimization. Only 4% of children were consistently victimized by peers, compared to 16% who were consistently victimized by siblings. Overall, the mental health outcomes in this sample deteriorated between age 11 and age 14 (i.e., self-esteem, life satisfaction, and body image all worsened).

**Table 1:** Descriptive statistics for victimization experiences, depressive symptoms, life satisfaction, self-esteem, and body image (*n* = 13, 912)

Exposures	Consistently low		Increasing		Decreasing		Consistently high		No siblings	
	*n*	% (99% CI)	*n*	% (99% CI)	*n*	% (99% CI)	*n*	% (99% CI)	*n*	% (99% CI)
Victimization by peers	10,732	77.14 (75.93, 78.36)	927	6.66 (5.96, 7.37)	1668	11.99 (11.02, 12.96)	584	4.20 (3.53, 4.87)	–	–
Victimization by siblings	6092	43.79 (42.43, 45.16)	1279	9.19 (8.39, 10.00)	3265	23.47 (22.23, 24.71)	2165	15.56 (14.50, 16.62)	1112	7.99 (7.31, 8.66)

Outcomes	Age 11		Age 14		*r*			
	Mean	(99% CI)	Mean	(99% CI)				
Depressive symptoms^a^	5.81	(5.74, 5.88)	5.63	(5.45, 5.81)	0.25			
Life satisfaction^a^	1.95	(1.90, 1.99)	2.47	(2.43, 2.52)	0.19			
Self-esteem^b^	16.85	(16.78, 16.92)	15.50	(15.41, 15.60)	0.29			
Body image^a^	2.52	(2.47, 2.57)	3.23	(3.18, 3.28)	0.28			

Descriptive statistics are based on the full sample, using the imputed dataset and with survey weights applied; depressive symptoms are measured on different scales at the two time-points, so the means here are not directly comparable; *r* shows correlation between outcomes between age 11 and age 14

^a^Higher scores = more symptoms/less satisfaction

^b^Higher scores = higher esteem

### Preliminary analyses

Significant victimization group * sex interaction terms in the preliminary regression models showed differences in the associations of interest between males and females (see Supplementary Tables S2 and S3). Consequently, all models were run separately for males and females. We also explored whether there was a multiplicative effect of being victimized by both peers and siblings by entering victimization by peers * victimization by siblings’ interaction terms in these models. None of the interaction terms were significant (see Supplementary Tables S4 and S5) and so only models with the main effects are presented here.

### Main models testing the association between peer and sibling victimization on mental health outcomes

Results of the final regression models are shown in Table 2 (for mental health outcomes at age 14) and Table 3 (for change in mental health outcomes between ages 11 and 14). When compared with those who experienced consistently low victimization, those who experienced consistently high victimization reported significantly poorer mental health-related outcomes at age 14, and significantly greater deterioration in mental health-related outcomes between ages 11 and 14, across all domains. This was the case for both sources of victimization, although the effect sizes for victimization by peers were larger than those for siblings. The only exception was changes in self-esteem in males between ages 11 and 14, in which there was no significant difference between those who experienced consistently high victimization and those who experience consistently low victimization. There was broadly the same pattern of results in males compared with females, although the coefficients were generally larger for females.

**Table 2:** Linear regression models showing the association between changes in victimization by peers and siblings and depressive symptoms, life satisfaction, self-esteem, and body image at age 14 (*n* = 13, 912)

	Depressive symptoms^a^		Life satisfaction^a^		Self-esteem^b^		Body image^a^	
	Males	Females	Males	Females	Males	Females	Males	Females
	*β* [99% CI]	*β* [99% CI]	*β* [99% CI]	*β* [99% CI]	*β* [99% CI]	*β* [99% CI]	*β* [99% CI]	*β* [99% CI]
Peers								
Consistently low	Ref	Ref	Ref	Ref	Ref	Ref	Ref	Ref
Increasing	0.603**	0.984**	0.470**	0.745**	− 0.375**	− 0.643**	0.395**	0.604**
	[0.465, 0.741]	[0.796, 1.171]	[0.329, 0.612]	[0.570, 0.921]	[− 0.551, − 0.199]	[− 0.804, − 0.482]	[0.233, 0.556]	[0.444, 0.764]
Decreasing	0.145*	0.254**	0.164*	0.218**	− 0.077	− 0.147*	0.146*	0.137*
	[0.022, 0.268]	[0.110, 0.397]	[0.027, 0.301]	[0.072, 0.364]	[− 0.198, 0.044]	[− 0.279, − 0.016]	[0.029, 0.263]	[0.000, 0.274]
Consistently high	0.914**	1.148**	0.692**	0.841**	− 0.430**	− 0.639**	0.487**	0.656**
	[0.680, 1.147]	[0.884, 1.412]	[0.466, 0.919]	[0.571, 1.112]	[− 0.652, − 0.209]	[− 0.876, − 0.403]	[0.264, 0.711]	[0.401, 0.912]
Siblings								
Consistently low	Ref	Ref	Ref	Ref	Ref	Ref	Ref	Ref
Increasing	0.205**	0.266**	0.197**	0.206**	− 0.157	− 0.168*	0.118	0.183**
	[0.070, 0.340]	[0.133, 0.399]	[0.064, 0.330]	[0.067, 0.344]	[− 0.318, 0.003]	[− 0.302, − 0.034]	[− 0.028, 0.265]	[0.051, 0.315]
Decreasing	0.088	0.022	0.062	0.028	− 0.052	− 0.070	0.040	0.060
	[− 0.005, 0.182]	[− 0.093, 0.138]	[− 0.035, 0.159]	[− 0.089, 0.146]	[− 0.149, 0.045]	[− 0.169, 0.030]	[− 0.082, 0.162]	[− 0.046, 0.166]
Consistently high	0.299**	0.299**	0.249**	0.284**	− 0.137*	− 0.240**	0.190**	0.233**
	[0.186, 0.411]	[0.177, 0.421]	[0.135, 0.364]	[0.162, 0.406]	[− 0.258, − 0.016]	[− 0.354, − 0.127]	[0.067, 0.313]	[0.119, 0.346]
No siblings	0.113	0.128	0.073	0.104	0.022	-0.040	0.070	0.084
	[− 0.031, 0.258]	[− 0.024, 0.281]	[− 0.067, 0.212]	[− 0.051, 0.259]	[− 0.120, 0.163]	[− 0.185, 0.105]	[− 0.073, 0.212]	[− 0.075, 0.243]

All models are adjusted for age, sex, ethnicity, family income, living in a two-parent household, being an only child, age 8 emotional problems, special educational needs, long-term illness, age 11 BMI percentile, BMI percentile change between age 11 and age 14, and pubertal development

*p < 0.01

**p < 0.001

^a^Positive coefficients indicate worse outcomes

^b^Positive coefficients indicate better outcomes

**Table 3:** Linear regression models showing the association between changes in victimization by peers and siblings and changes in depressive symptoms, life satisfaction, self-esteem, and body image between ages 11 and 14 (n = 13, 912)

	Depressive symptoms^a^		Life satisfaction^a^		Self-esteem^b^		Body image^a^	
	Males	Females	Males	Females	Males	Females	Males	Females
	*β* [99% CI]	*β* [99% CI]	*β* [99% CI]	*β* [99% CI]	*β* [99% CI]	*β* [99% CI]	*β* [99% CI]	*β* [99% CI]
Baseline level of outcome	0.133**	0.207**	0.139**	0.160**	0.241**	0.248**	0.202**	0.217**
	[0.093, 0.173]	[0.160, 0.254]	[0.101, 0.178]	[0.116, 0.203]	[0.202, 0.280]	[0.207, 0.289]	[0.159, 0.244]	[0.179, 0.256]
Peers								
Consistently low	Ref	Ref	Ref	Ref	Ref	Ref	Ref	Ref
Increasing	0.570**	0.928**	0.446**	0.726**	− 0.333**	− 0.614**	0.356**	0.568**
	[0.432, 0.708]	[0.744, 1.113]	[0.308, 0.584]	[0.553, 0.898]	[− 0.502, − 0.164]	[− 0.773, − 0.456]	[0.198, 0.514]	[0.408, 0.728]
Decreasing	0.038	0.076	0.115	0.149*	0.001	− 0.038	0.087	0.036
	[− 0.088, 0.164]	[− 0.066, 0.217]	[− 0.021, 0.250]	[0.002, 0.296]	[− 0.114, 0.115]	[− 0.164, 0.087]	[− 0.028, 0.203]	[− 0.098, 0.170]
Consistently high	0.785**	0.890**	0.616**	0.749**	− 0.294**	− 0.480**	0.383**	0.514**
	[0.560, 1.011]	[0.625, 1.155]	[0.389, 0.844]	[0.493, 1.006]	[− 0.506, − 0.083]	[− 0.718, − 0.242]	[0.164, 0.601]	[0.261, 0.767]
Siblings								
Consistently low	Ref	Ref	Ref	Ref	Ref	Ref	Ref	Ref
Increasing	0.198**	0.264**	0.192**	0.202**	− 0.148	− 0.171*	0.115	0.176**
	[0.065, 0.331]	[0.130, 0.398]	[0.061, 0.322]	[0.066, 0.338]	[− 0.306, 0.010]	[− 0.305, − 0.036]	[− 0.027, 0.256]	[0.046, 0.306]
Decreasing	0.059	− 0.045	0.036	− 0.001	− 0.007	− 0.019	0.003	0.022
	[− 0.033, 0.151]	[− 0.160, 0.071]	[− 0.062, 0.134]	[− 0.113, 0.112]	[− 0.103, 0.088]	[− 0.115, 0.077]	[− 0.117, 0.123]	[− 0.080, 0.123]
Consistently high	0.256**	0.236**	0.223**	0.252**	− 0.107	− 0.194**	0.161**	0.186**
	[0.146, 0.367]	[0.117, 0.354]	[0.112, 0.335]	[0.131, 0.374]	[− 0.223, 0.008]	[− 0.307, − 0.082]	[0.042, 0.280]	[0.074, 0.299]
No siblings	0.092	0.124	0.070	0.095	0.026	− 0.029	0.070	0.070
	[− 0.051, 0.235]	[− 0.025, 0.273]	[− 0.066, 0.207]	[− 0.058, 0.249]	[− 0.116, 0.169]	[− 0.171, 0.112]	[− 0.072, 0.212]	[− 0.089, 0.228]

All models are adjusted for age, sex, ethnicity, family income, living in a two-parent household, being an only child, age 8 emotional problems, special educational needs, long-term illness, age 11 BMI percentile, BMI percentile change between age 11 and age 14, and pubertal development

*p < 0.01

**p < 0.001

^a^positive coefficients indicate greater deterioration in outcomes

^b^positive coefficients indicate greater improvement outcomes

Turning to the increasing victimization group, these participants also reported significantly worse mental health-related outcomes at age 14, and significantly greater deterioration in mental health outcomes between ages 11 and 14, compared with the consistently low group. Again, the only exception was levels of self-esteem in males in relation to sibling victimization (both at age 14 and changes between ages 11 and 14). The 99% CIs show that the mental health outcomes at age 14 of the increasing victimization group were indistinguishable from those in the consistently high victimization group. Interestingly, the amount of change in mental health outcomes between ages 11 and 14 were also similar in these two groups (increasing and consistently high). Once again, the effect sizes were larger for victimization by peers than siblings, and for females compared with males.

Finally, those who experienced decreasing victimization by siblings showed no differences in mental health-related outcomes at age 14, or changes in mental health outcomes between ages 11 and 14, compared with the consistently low group. In contrast, the decreasing victimization by peers group did show significantly worse mental health-related outcomes at age 14 compared with the consistently low group, although the differences were much less marked than for the consistently high or increasing group. The decreasing victimization by peers groups did not show any differences in changes in mental health outcomes between age 11 and age 14 compared with the consistently low group, with the exception of for life satisfaction in females.

## Discussion

Using longitudinal data from a large national prospective birth cohort study, this paper investigated how changes in peer and sibling victimization are associated with changes in multiple indices of mental health between 11 and 14 years of age. The results clearly show that any experience of victimization is associated with substantially poorer mental health, both concurrently and 3 years later. This was demonstrated in all three victimization profiles (e.g., consistently high, and increasing and decreasing victimization), for whom decreased mental health outcomes were reported in comparison to the group who did not experience victimization at any time point. In line with our hypotheses, the poorest mental health outcomes were found for the minority of children (4–16%) who had consistently high victimization experiences: these children have both poorer mental health at age 14 and demonstrate a greater deterioration in symptoms between ages 11 and 14 years compared to their peers. This suggests an ongoing and cumulative impact of being consistently victimized on young people’s mental health and well-being.

Around 12% children experienced a decreasing pattern of victimization from peers and 23% from siblings (i.e., were victimized in primary school but not in secondary school). Importantly, those who experienced decreasing victimization in this period had better mental health outcomes by age 14 than those who were consistently victimized, but worse than those who were not victimized. Moreover, they showed similar rates of change in mental health outcomes as those who were not victimized. Taken together, these findings highlight both the enduring effect of victimization from peers on mental health and, notably, the potential for young people to regain a rate of change in mental health symptoms that is akin to their non-victimized peers when victimization is no longer being experienced. It is important to note here that we operationalised experiences of victimization based on frequency of at least once a week, and although this provides a useful marker of frequent victimization that can be targets of intervention, these analyses do not provide information regarding the potential impacts on mental health of changes in less frequent or rare victimization.

Peer and sibling victimization were shown to have independent, additive associations with mental health outcomes, consistent with previous research in this area [3, 21]. This highlights the role of victimization by siblings for mental health, which we found to be more frequent than peer victimization, yet is often overlooked by parents, prevention programmes, and research [39]. Nonetheless, while the observed directions of associations were similar for peer and sibling victimization, we found consistently larger effects for peer victimization compared to sibling victimization. This may be due to the fact that up to 85% of peer victimization takes place in the presence of classmates, either supporting the victimizer or failing to support the victim, potentially adding a level of social shame and humiliation to the victimization experience, which can be contrasted with sibling victimization which is more likely to take place in the context of the dyadic sibling relationship [40].

Interestingly, there was no multiplicative effect between peer and sibling victimization, that is, the impact of peer victimization was not magnified in the presence of sibling victimization. Our finding is consistent with the small amount of other research which has specifically tested for multiplicative effects of peer and sibling victimization [39]. It also aligns well with other work showing the cumulative effect of victimization across multiple environments (e.g., in school and online) on mental health [41].

Collectively, these findings highlight the potential for bullying prevention and intervention programmes to impact on youth mental health. Peer bullying prevention and reduction interventions have evidence of efficacy [42], yet only approximately half of schools in an international survey of school provision report having substantial anti-bullying provision in their schools [43]. Our findings indicate that stopping or reducing experiences of peer victimization may prevent a deterioration of their mental health. Examining these mental health outcomes over a longer term in future anti-bullying interventions would be valuable to assess whether these findings are mirrored in intervention settings. With regards to sibling victimization, our findings indicate that although it is a common experience, it is not harmless. Greater awareness by both parents and clinicians of the consequences of sibling victimization would be beneficial, and clinicians should widen questions around victimization experiences of their clients to include siblings. Development and rigorous evaluation of sibling bullying prevention programmes is clearly warranted.

Strengths of the current study include the use of a large, nationally representative cohort study and multiple indices of child mental health. Furthermore, the repeated measures of bullying and mental health enabled us to examine whether changes in bullying are associated with changes in mental health outcomes [44].

This study also demonstrates a number of limitations. First, both peer and sibling victimization were assessed with a single item with a direct behavioural anchor (i.e., ‘how often do other children/siblings hurt or pick on you on purpose’). In this item, peer victimization is operationalised through reference to ‘other children’ and so it is plausible that participants may have responded in relation to siblings as well as peers. It should be noted, however, that the broad group sizes categorizing changing sibling victimization align with work by Tucker and colleagues [15, 19] using a comprehensive index of victimization, suggesting accuracy in participant report of sibling bullying using the single item employed in the current study.

It was also not possible to explore which sibling (e.g., relative age, gender) was the perpetrator of bullying. While using single items of this kind a common approach in large cohort studies [45, 46], it does preclude a more-nuanced examination of different forms of victimization (e.g., direct compared to indirect victimization) that may have differential impacts on young people’s mental health, as well as direct comparisons to studies using more comprehensive measures of bullying, including features such as power imbalance and repetition by the same perpetrator. This is a broader problem in the extant literature and highlights the need to develop reliable and validated measures of bullying behaviour that are appropriate for use in large cohort studies.

Second, given the large sample size, we were unable to corroborate the results of the self-report mental health measures with clinical interviews or formal diagnoses. Third, although a large number of potential confounds were controlled in the statistical models (e.g., ethnicity, family income, living in a two-parent household, and earlier emotional problems), it remains plausible that there are other confounding factors that might explain the observed findings by both predisposing children to victimization and also increase their risk of mental health difficulties [7]. Finally, the analysis was limited to two time-points. Whilst these spanned a key developmental period of transition, analysis of further time-points would allow more-nuanced exploration of the trajectories of change in both victimization and mental health outcomes.

In conclusion, this study employs data from a national longitudinal cohort of children transitioning from early to mid-adolescence to investigate changes in the experiences of peer and sibling victimization and how these relate to changes in depressive symptoms, self-esteem, body satisfaction, and subjective well-being. We find that victimization at any time point is associated with poorer mental health outcomes, with this being particularly marked for those who experience consistent victimization over time and in both home and school contexts. Moreover, for young people whose victimization from peers and siblings decreases over these years, their deterioration in mental health outcomes is substantially smaller than for those with persistently high or increasing victimization experiences. These findings have promising implications for bullying interventions at an age where adolescents are highly susceptible to mental health difficulties with lifelong consequences.

## Supplementary Information

Below is the link to the electronic supplementary material.

Supplementary file1 (DOCX 71 KB)

## Data Availability

http://www.cls.ioe.ac.uk.
