# Supplementary material for: Changes in peer and sibling victimization in early adolescence: longitudinal associations with multiple indices of mental health in a prospective birth cohort study
Source: Eur Child Adolesc Psychiatry. 2021 Jan 11;31(5):737–46. doi: 10.1007/s00787-020-01708-z (PMC9142430; doi:10.1007/s00787-020-01708-z)
Supplement: Supplementary file 1 — Supplementary file1 (DOCX 71 KB) [file 787_2020_1708_MOESM1_ESM.docx]

**Table S1. Original distribution of responses to victimization items: “How often other children hurt or pick on you on purpose” (peers) and “How often your brothers or sisters hurt or pick on you on purpose” (siblings), (n = 13, 912)**

|  | Victimization by peers | | Victimization by Siblings | |
| --- | --- | --- | --- | --- |
| Response options | Age 11 | Age 14 | Age 11 | Age14 |
| Most days | 6.85% | 4.60% | 22.21% | 12.12% |
| About once a week | 9.14% | 6.22% | 20.53% | 15.05% |
| About once a month | 7.19% | 5.24% | 7.21% | 7.00% |
| Every few months | 7.04% | 5.26% | 6.03% | 4.72% |
| Less often | 27.23% | 27.81% | 20.68% | 24.38% |
| Never | 42.55% | 50.87% | 23.34% | 36.73% |

Table S2. Linear regression models showing sex interactions in the associations between changes in victimization by peers and siblings and depressive symptoms, life satisfaction, self-esteem and body image at age 14 (n = 13, 912)

|  | Depressive symptoms  β [99% CI] | Life satisfaction  β [99% CI] | Self esteem  β [99% CI] | Body image  β [99% CI] |
| --- | --- | --- | --- | --- |
| Sex | 0.374** | 0.282** | -0.462** | 0.497** |
|  | [0.286,0.463] | [0.187,0.376] | [-0.551,-0.373] | [0.400,0.593] |
| Peers: |  |  |  |  |
| Increasing | 0.607** | 0.474** | -0.382** | 0.401** |
|  | [0.467,0.746] | [0.333,0.616] | [-0.559,-0.206] | [0.240,0.562] |
| Decreasing | 0.143* | 0.160* | -0.077 | 0.145* |
|  | [0.021,0.264] | [0.025,0.295] | [-0.198,0.044] | [0.028,0.262] |
| Consistently high | 0.910** | 0.686** | -0.433** | 0.492** |
|  | [0.678,1.142] | [0.460,0.912] | [-0.653,-0.213] | [0.268,0.716] |
| Increasing * Sex | 0.383** | 0.278* | -0.261* | 0.202 |
|  | [0.154,0.612] | [0.052,0.503] | [-0.503,-0.019] | [-0.022,0.426] |
| Decreasing * Sex | 0.11 | 0.065 | -0.07 | -0.008 |
|  | [-0.082,0.303] | [-0.127,0.258] | [-0.238,0.099] | [-0.188,0.172] |
| Consistently high * Sex | 0.238 | 0.162 | -0.201 | 0.158 |
|  | [-0.099,0.576] | [-0.173,0.497] | [-0.497,0.094] | [-0.162,0.479] |
| Siblings: |  |  |  |  |
| Increasing | 0.210** | 0.200** | -0.162* | 0.122 |
|  | [0.074,0.346] | [0.066,0.333] | [-0.322,-0.002] | [-0.023,0.268] |
| Decreasing | 0.09 | 0.063 | -0.054 | 0.041 |
|  | [-0.004,0.183] | [-0.034,0.160] | [-0.152,0.043] | [-0.080,0.163] |
| Consistently high | 0.307** | 0.254** | -0.146* | 0.196** |
|  | [0.196,0.419] | [0.138,0.369] | [-0.267,-0.024] | [0.074,0.319] |
| No siblings | 0.094 | 0.068 | 0.029 | 0.062 |
|  | [-0.049,0.238] | [-0.068,0.205] | [-0.112,0.171] | [-0.078,0.202] |
| Increasing * Sex | 0.052 | 0.010 | -0.005 | 0.065 |
|  | [-0.142,0.246] | [-0.191,0.211] | [-0.209,0.199] | [-0.135,0.266] |
| Decreasing * Sex | -0.072 | -0.036 | -0.012 | 0.017 |
|  | [-0.220,0.076] | [-0.188,0.116] | [-0.151,0.127] | [-0.141,0.175] |
| Consistently high * Sex | -0.012 | 0.032 | -0.093 | 0.038 |
|  | [-0.183,0.159] | [-0.128,0.191] | [-0.256,0.070] | [-0.124,0.200] |
| No siblings * Sex | 0.051 | 0.038 | -0.075 | 0.030 |
|  | [-0.143,0.244] | [-0.161,0.237] | [-0.269,0.120] | [-0.177,0.236] |

Note. * p < 0.01, ** p < 0.001. All models are adjusted for age, sex, ethnicity, family income, living in a two-parent household, being an only child, age 8 emotional problems, special educational needs, long term illness, age 11 BMI percentile, BMI percentile change between age 11 and age 14, and pubertal development.

Table S3. Linear regression models showing sex interactions in the associations between changes in victimization by peers and siblings and changes in depressive symptoms, life satisfaction, self-esteem and body image between ages 11 and age 14 (n = 13, 912)

|  | Depressive symptoms  β [99% CI] | Life satisfaction  β [99% CI] | Self esteem  β [99% CI] | Body image  β [99% CI] |
| --- | --- | --- | --- | --- |
| Sex | 0.357** | 0.283** | -0.433** | 0.466** |
|  | [0.270,0.443] | [0.188,0.378] | [-0.519,-0.346] | [0.372,0.559] |
| Baseline level of outcome | 0.169** | 0.149** | 0.245** | 0.210** |
|  | [0.137,0.202] | [0.121,0.177] | [0.216,0.275] | [0.183,0.238] |
| Peers: |  |  |  |  |
| Increasing | 0.564** | 0.449** | -0.340** | 0.361** |
|  | [0.424,0.704] | [0.311,0.587] | [-0.510,-0.170] | [0.205,0.518] |
| Decreasing | 0.006 | 0.107 | 0.002 | 0.083 |
|  | [-0.119,0.130] | [-0.026,0.240] | [-0.112,0.117] | [-0.032,0.198] |
| Consistently high | 0.746** | 0.606** | -0.295** | 0.382** |
|  | [0.524,0.968] | [0.381,0.831] | [-0.505,-0.085] | [0.163,0.602] |
| Increasing * Sex | 0.381** | 0.284** | -0.273* | 0.207 |
|  | [0.154,0.608] | [0.064,0.505] | [-0.511,-0.036] | [-0.015,0.428] |
| Decreasing * Sex | 0.102 | 0.053 | -0.041 | -0.044 |
|  | [-0.083,0.288] | [-0.135,0.242] | [-0.203,0.121] | [-0.218,0.129] |
| Consistently high * Sex | 0.192 | 0.157 | -0.18 | 0.13 |
|  | [-0.133,0.518] | [-0.172,0.486] | [-0.471,0.111] | [-0.189,0.449] |
| Siblings: |  |  |  |  |
| Increasing | 0.201** | 0.194** | -0.153 | 0.118 |
|  | [0.068,0.334] | [0.064,0.325] | [-0.310,0.004] | [-0.023,0.259] |
| Decreasing | 0.052 | 0.035 | -0.009 | 0.003 |
|  | [-0.040,0.144] | [-0.062,0.133] | [-0.104,0.086] | [-0.115,0.120] |
| Consistently high | 0.253** | 0.226** | -0.115 | 0.165** |
|  | [0.144,0.363] | [0.114,0.338] | [-0.231,0.000] | [0.047,0.284] |
| No siblings | 0.067 | 0.065 | 0.032 | 0.064 |
|  | [-0.074,0.208] | [-0.068,0.198] | [-0.110,0.174] | [-0.075,0.203] |
| Increasing * Sex | 0.059 | 0.013 | -0.017 | 0.063 |
|  | [-0.133,0.251] | [-0.184,0.210] | [-0.217,0.183] | [-0.133,0.259] |
| Decreasing * Sex | -0.089 | -0.035 | -0.007 | 0.019 |
|  | [-0.236,0.059] | [-0.185,0.114] | [-0.142,0.128] | [-0.135,0.173] |
| Consistently high * Sex | -0.01 | 0.03 | -0.078 | 0.024 |
|  | [-0.174,0.155] | [-0.127,0.187] | [-0.238,0.082] | [-0.134,0.183] |
| No siblings * Sex | 0.074 | 0.033 | -0.066 | 0.012 |
|  | [-0.115,0.263] | [-0.164,0.231] | [-0.255,0.124] | [-0.194,0.217] |

Note. * p < 0.01, ** p < 0.001. All models are adjusted for age, sex, ethnicity, family income, living in a two-parent household, being an only child, age 8 emotional problems, special educational needs, long term illness, age 11 BMI percentile, BMI percentile change between age 11 and age 14, and pubertal development.

Table S4. Linear regression models showing peer * sibling interactions in the associations between changes in victimization by peers and siblings and depressive symptoms, life satisfaction, self-esteem and body image at age 14 (n = 13, 912)

|  | Males | | | | Females | | | |
| --- | --- | --- | --- | --- | --- | --- | --- | --- |
|  | Depressive symptoms  β [99% CI] | Life satisfaction  β [99% CI] | Self esteem  β [99% CI] | Body image  β [99% CI] | Depressive symptoms  β [99% CI] | Life satisfaction  β [99% CI] | Self esteem  β [99% CI] | Body image  β [99% CI] |
| Peers: |  |  |  |  |  |  |  |  |
| Increasing | 0.604** | 0.488** | -0.29 | 0.479** | 1.155** | 0.967** | -0.818** | 0.708** |
|  | [0.331,0.878] | [0.207,0.769] | [-0.580,0.000] | [0.150,0.808] | [0.749,1.561] | [0.597,1.337] | [-1.246,-0.389] | [0.343,1.073] |
| Decreasing | 0.17 | 0.222 | -0.162 | 0.163 | 0.342* | 0.357* | -0.132 | 0.245 |
|  | [-0.047,0.388] | [-0.012,0.455] | [-0.387,0.063] | [-0.062,0.388] | [0.035,0.648] | [0.060,0.654] | [-0.400,0.137] | [-0.012,0.501] |
| Consistently high | 1.044** | 0.752** | -0.580** | 0.683** | 0.944** | 0.625** | -0.491 | 0.621* |
|  | [0.545,1.543] | [0.283,1.220] | [-0.985,-0.174] | [0.229,1.137] | [0.458,1.431] | [0.186,1.063] | [-1.032,0.049] | [0.106,1.137] |
| Siblings: |  |  |  |  |  |  |  |  |
| Increasing | 0.220** | 0.211** | -0.184* | 0.156* | 0.264** | 0.246** | -0.164* | 0.203** |
|  | [0.074,0.367] | [0.063,0.359] | [-0.356,-0.011] | [0.000,0.312] | [0.117,0.411] | [0.098,0.394] | [-0.308,-0.021] | [0.064,0.342] |
| Decreasing | 0.094 | 0.077 | -0.065 | 0.05 | 0.043 | 0.058 | -0.075 | 0.081 |
|  | [-0.009,0.198] | [-0.039,0.193] | [-0.171,0.041] | [-0.088,0.188] | [-0.083,0.169] | [-0.064,0.181] | [-0.177,0.026] | [-0.035,0.197] |
| Consistently high | 0.325** | 0.281** | -0.171* | 0.240** | 0.333** | 0.322** | -0.249** | 0.259** |
|  | [0.186,0.464] | [0.143,0.419] | [-0.315,-0.026] | [0.091,0.389] | [0.202,0.464] | [0.186,0.458] | [-0.384,-0.114] | [0.131,0.387] |
| No siblings | 0.117 | 0.064 | 0.039 | 0.073 | 0.142 | 0.092 | -0.067 | 0.091 |
|  | [-0.039,0.274] | [-0.084,0.212] | [-0.127,0.205] | [-0.084,0.230] | [-0.030,0.314] | [-0.094,0.278] | [-0.224,0.090] | [-0.087,0.269] |
| Peers * siblings interaction |  |  |  |  |  |  |  |  |
| Peers: Increasing * Siblings: Increasing | -0.014 | -0.039 | -0.041 | -0.183 | -0.124 | -0.26 | 0.143 | -0.068 |
|  | [-0.494,0.467] | [-0.477,0.400] | [-0.492,0.410] | [-0.699,0.333] | [-0.672,0.424] | [-0.757,0.237] | [-0.391,0.677] | [-0.547,0.411] |
| Peers: Increasing * Siblings: Decreasing | 0.128 | 0.049 | -0.152 | 0.043 | -0.121 | -0.307 | 0.163 | -0.056 |
|  | [-0.327,0.583] | [-0.467,0.566] | [-0.642,0.339] | [-0.477,0.563] | [-0.791,0.550] | [-0.858,0.243] | [-0.512,0.838] | [-0.657,0.545] |
| Peers: Increasing * Siblings: Consistently high | -0.075 | -0.129 | -0.059 | -0.255 | -0.335 | -0.344 | 0.298 | -0.227 |
|  | [-0.490,0.339] | [-0.559,0.302] | [-0.465,0.347] | [-0.683,0.173] | [-0.861,0.191] | [-0.813,0.125] | [-0.224,0.820] | [-0.701,0.247] |
| Peers: Increasing * Siblings: No siblings | -0.086 | 0.115 | -0.347 | -0.024 | -0.37 | -0.31 | 0.379 | -0.222 |
|  | [-0.762,0.590] | [-0.549,0.779] | [-0.981,0.288] | [-0.656,0.608] | [-1.081,0.341] | [-0.973,0.353] | [-0.356,1.115] | [-0.860,0.417] |
| Peers: Decreasing * Siblings: Increasing | 0.035 | 0.087 | 0.06 | -0.037 | -0.108 | -0.438 | -0.055 | -0.263 |
|  | [-0.452,0.523] | [-0.529,0.703] | [-0.536,0.656] | [-0.629,0.556] | [-0.841,0.625] | [-1.033,0.156] | [-0.749,0.640] | [-0.930,0.405] |
| Peers: Decreasing * Siblings: Decreasing | -0.06 | -0.13 | 0.143 | -0.062 | -0.189 | -0.247 | 0.014 | -0.222 |
|  | [-0.316,0.195] | [-0.411,0.152] | [-0.136,0.421] | [-0.353,0.229] | [-0.582,0.203] | [-0.622,0.127] | [-0.329,0.357] | [-0.554,0.110] |
| Peers: Decreasing * Siblings: Consistently high | -0.047 | -0.043 | 0.135 | 0.034 | -0.07 | -0.172 | -0.054 | -0.083 |
|  | [-0.380,0.286] | [-0.416,0.330] | [-0.207,0.477] | [-0.339,0.407] | [-0.462,0.321] | [-0.544,0.199] | [-0.424,0.315] | [-0.437,0.271] |
| Peers: Decreasing * Siblings: No siblings | 0.077 | -0.056 | 0.011 | 0.018 | -0.087 | 0.115 | 0.003 | -0.012 |
|  | [-0.410,0.564] | [-0.685,0.572] | [-0.454,0.476] | [-0.470,0.507] | [-0.685,0.512] | [-0.512,0.742] | [-0.603,0.609] | [-0.550,0.525] |
| Peers: Consistently high * Siblings: Increasing | -0.421 | -0.412 | 0.48 | -0.427 | 0.378 | 0.134 | -0.186 | -0.12 |
|  | [-1.240,0.398] | [-1.102,0.278] | [-0.298,1.259] | [-1.241,0.387] | [-0.585,1.341] | [-0.878,1.147] | [-1.040,0.668] | [-1.114,0.875] |
| Peers: Consistently high * Siblings: Decreasing | -0.102 | 0.044 | 0.058 | -0.095 | 0.326 | 0.424 | -0.239 | 0.249 |
|  | [-0.762,0.558] | [-0.559,0.647] | [-0.519,0.635] | [-0.698,0.508] | [-0.424,1.076] | [-0.401,1.249] | [-1.024,0.546] | [-0.503,1.000] |
| Peers: Consistently high * Siblings: Consistently high | -0.182 | -0.143 | 0.255 | -0.375 | 0.101 | 0.165 | -0.167 | -0.035 |
|  | [-0.734,0.370] | [-0.706,0.419] | [-0.233,0.743] | [-0.948,0.198] | [-0.496,0.699] | [-0.384,0.714] | [-0.787,0.454] | [-0.643,0.572] |
| Peers: Consistently high * Siblings: No siblings | -0.192 | 0.083 | 0.181 | -0.205 | 0.499 | 0.367 | 0.003 | 0.114 |
|  | [-0.935,0.550] | [-0.869,1.034] | [-0.544,0.907] | [-0.999,0.589] | [-0.368,1.366] | [-0.486,1.219] | [-1.007,1.012] | [-0.848,1.075] |

Note. * p < 0.01, ** p < 0.001. All models are adjusted for age, sex, ethnicity, family income, living in a two-parent household, being an only child, age 8 emotional problems, special educational needs, long term illness, age 11 BMI percentile, BMI percentile change between age 11 and age 14, and pubertal development.

Table S5. Linear regression models showing peer * sibling interactions in the associations between changes in victimization by peers and siblings and changes in depressive symptoms, life satisfaction, self-esteem and body image between ages 11 and age 14 (n = 13, 912)

|  | Males | | | | Females | | | |
| --- | --- | --- | --- | --- | --- | --- | --- | --- |
|  | Depressive symptoms  β [99% CI] | Life satisfaction  β [99% CI] | Self esteem  β [99% CI] | Body image  β [99% CI] | Depressive symptoms  β [99% CI] | Life satisfaction  β [99% CI] | Self esteem  β [99% CI] | Body image  β [99% CI] |
| Baseline level of outcome | 0.133** | 0.139** | 0.241** | 0.202** | 0.207** | 0.159** | 0.248** | 0.217** |
|  | [0.092,0.173] | [0.100,0.177] | [0.203,0.280] | [0.159,0.244] | [0.159,0.254] | [0.116,0.202] | [0.207,0.290] | [0.178,0.256] |
| Peers: |  |  |  |  |  |  |  |  |
| Increasing | 0.566** | 0.454** | -0.255 | 0.452** | 1.075** | 0.938** | -0.777** | 0.636** |
|  | [0.291,0.841] | [0.175,0.733] | [-0.551,0.042] | [0.137,0.767] | [0.684,1.466] | [0.574,1.302] | [-1.186,-0.368] | [0.278,0.994] |
| Decreasing | 0.041 | 0.161 | -0.08 | 0.08 | 0.141 | 0.275 | 0.013 | 0.13 |
|  | [-0.175,0.257] | [-0.070,0.392] | [-0.302,0.143] | [-0.135,0.295] | [-0.150,0.432] | [-0.012,0.563] | [-0.235,0.261] | [-0.121,0.381] |
| Consistently high | 0.894** | 0.647** | -0.440* | 0.567** | 0.709** | 0.533* | -0.324 | 0.527* |
|  | [0.415,1.374] | [0.175,1.119] | [-0.814,-0.065] | [0.136,0.998] | [0.229,1.189] | [0.089,0.976] | [-0.803,0.154] | [0.052,1.002] |
| Siblings: |  |  |  |  |  |  |  |  |
| Increasing | 0.210** | 0.207** | -0.176* | 0.15 | 0.262** | 0.244** | -0.162* | 0.194** |
|  | [0.064,0.357] | [0.061,0.353] | [-0.349,-0.003] | [-0.001,0.302] | [0.117,0.406] | [0.098,0.390] | [-0.305,-0.018] | [0.058,0.330] |
| Decreasing | 0.059 | 0.046 | -0.016 | 0.008 | -0.029 | 0.028 | -0.024 | 0.039 |
|  | [-0.042,0.159] | [-0.071,0.162] | [-0.119,0.086] | [-0.127,0.144] | [-0.155,0.097] | [-0.092,0.147] | [-0.122,0.074] | [-0.075,0.152] |
| Consistently high | 0.277** | 0.247** | -0.143* | 0.200** | 0.265** | 0.286** | -0.201** | 0.212** |
|  | [0.138,0.415] | [0.110,0.383] | [-0.285,-0.001] | [0.056,0.345] | [0.134,0.396] | [0.152,0.420] | [-0.333,-0.069] | [0.085,0.339] |
| No siblings | 0.094 | 0.062 | 0.038 | 0.078 | 0.132 | 0.081 | -0.032 | 0.069 |
|  | [-0.060,0.247] | [-0.084,0.208] | [-0.128,0.203] | [-0.076,0.232] | [-0.037,0.301] | [-0.102,0.264] | [-0.183,0.119] | [-0.106,0.244] |
| Peers * siblings interaction |  |  |  |  |  |  |  |  |
| Peers: Increasing * Siblings: Increasing | 0.006 | -0.062 | 0.02 | -0.195 | -0.096 | -0.25 | 0.096 | -0.021 |
|  | [-0.473,0.486] | [-0.491,0.367] | [-0.440,0.479] | [-0.682,0.291] | [-0.645,0.454] | [-0.742,0.242] | [-0.422,0.613] | [-0.491,0.449] |
| Peers: Increasing * Siblings: Decreasing | 0.11 | 0.088 | -0.199 | 0.027 | -0.056 | -0.311 | 0.187 | -0.024 |
|  | [-0.348,0.569] | [-0.433,0.609] | [-0.691,0.292] | [-0.479,0.532] | [-0.731,0.618] | [-0.856,0.234] | [-0.487,0.862] | [-0.627,0.579] |
| Peers: Increasing * Siblings: Consistently high | -0.063 | -0.107 | -0.04 | -0.264 | -0.32 | -0.331 | 0.322 | -0.188 |
|  | [-0.474,0.348] | [-0.533,0.318] | [-0.454,0.374] | [-0.683,0.154] | [-0.826,0.185] | [-0.803,0.141] | [-0.185,0.828] | [-0.666,0.289] |
| Peers: Increasing * Siblings: No siblings | -0.06 | 0.142 | -0.344 | -0.052 | -0.304 | -0.259 | 0.249 | -0.118 |
|  | [-0.721,0.602] | [-0.498,0.782] | [-0.959,0.271] | [-0.641,0.537] | [-1.004,0.396] | [-0.904,0.386] | [-0.453,0.950] | [-0.729,0.494] |
| Peers: Decreasing * Siblings: Increasing | 0.05 | 0.097 | 0.003 | -0.011 | -0.1 | -0.466 | -0.037 | -0.285 |
|  | [-0.422,0.521] | [-0.511,0.704] | [-0.557,0.563] | [-0.575,0.553] | [-0.847,0.647] | [-1.050,0.119] | [-0.749,0.676] | [-0.923,0.352] |
| Peers: Decreasing * Siblings: Decreasing | -0.019 | -0.108 | 0.136 | -0.028 | -0.165 | -0.224 | -0.032 | -0.192 |
|  | [-0.272,0.234] | [-0.388,0.172] | [-0.141,0.413] | [-0.303,0.247] | [-0.546,0.216] | [-0.592,0.144] | [-0.357,0.293] | [-0.529,0.145] |
| Peers: Decreasing * Siblings: Consistently high | -0.018 | -0.019 | 0.123 | 0.091 | -0.024 | -0.144 | -0.099 | -0.067 |
|  | [-0.351,0.315] | [-0.381,0.343] | [-0.208,0.454] | [-0.276,0.457] | [-0.409,0.362] | [-0.513,0.225] | [-0.449,0.252] | [-0.415,0.282] |
| Peers: Decreasing * Siblings: No siblings | 0.079 | -0.071 | 0.061 | 0.013 | -0.063 | 0.109 | -0.101 | -0.008 |
|  | [-0.402,0.561] | [-0.697,0.556] | [-0.408,0.530] | [-0.466,0.493] | [-0.640,0.514] | [-0.495,0.713] | [-0.657,0.455] | [-0.521,0.505] |
| Peers: Consistently high * Siblings: Increasing | -0.43 | -0.337 | 0.422 | -0.407 | 0.309 | 0.133 | -0.179 | -0.165 |
|  | [-1.243,0.383] | [-1.001,0.327] | [-0.307,1.151] | [-1.178,0.364] | [-0.685,1.303] | [-0.891,1.157] | [-1.006,0.648] | [-1.151,0.820] |
| Peers: Consistently high * Siblings: Decreasing | -0.062 | 0.079 | 0.046 | -0.068 | 0.339 | 0.413 | -0.169 | 0.2 |
|  | [-0.715,0.591] | [-0.521,0.679] | [-0.507,0.598] | [-0.663,0.528] | [-0.379,1.057] | [-0.407,1.233] | [-0.905,0.567] | [-0.513,0.912] |
| Peers: Consistently high * Siblings: Consistently high | -0.146 | -0.097 | 0.264 | -0.348 | 0.071 | 0.171 | -0.188 | -0.104 |
|  | [-0.689,0.397] | [-0.667,0.472] | [-0.197,0.725] | [-0.903,0.207] | [-0.510,0.651] | [-0.375,0.717] | [-0.747,0.370] | [-0.685,0.476] |
| Peers: Consistently high * Siblings: No siblings | -0.169 | 0.084 | 0.184 | -0.254 | 0.473 | 0.384 | -0.155 | 0.105 |
|  | [-0.903,0.566] | [-0.859,1.028] | [-0.517,0.886] | [-1.054,0.545] | [-0.371,1.317] | [-0.501,1.269] | [-1.165,0.855] | [-0.852,1.063] |

Note. * p < 0.01, ** p < 0.001. All models are adjusted for age, sex, ethnicity, family income, living in a two-parent household, being an only child, age 8 emotional problems, special educational needs, long term illness, age 11 BMI percentile, BMI percentile change between age 11 and age 14, and pubertal development.

**Table S6. Sensitivity analysis: Linear regression models showing the association between changes in victimization by peers and siblings and depressive symptoms, life satisfaction, self-esteem and body image at age 14, excluding only children (n = 12,802)**

|  | Depressive symptoms ^a^ | | Life satisfaction ^a^ | | Self esteem ^b^ | | Body image ^a^ | |
| --- | --- | --- | --- | --- | --- | --- | --- | --- |
|  | Males | Females | Males | Females | Males | Females | Males | Females |
|  | β [99% CI] | β [99% CI] | β [99% CI] | β [99% CI] | β [99% CI] | β [99% CI] | β [99% CI] | β [99% CI] |
| Peers: |  |  |  |  |  |  |  |  |
| Consistently low | Ref. | Ref. | Ref. | Ref. | Ref. | Ref. | Ref. | Ref. |
| Increasing | 0.611** | 1.003** | 0.456** | 0.754** | -0.350** | -0.663** | 0.388** | 0.615** |
|  | [0.463,0.759] | [0.808,1.198] | [0.304,0.607] | [0.564,0.944] | [-0.536,-0.165] | [-0.833,-0.493] | [0.218,0.558] | [0.447,0.783] |
| Decreasing | 0.138* | 0.254** | 0.165* | 0.196* | -0.072 | -0.150* | 0.144* | 0.130 |
|  | [0.014,0.261] | [0.102,0.406] | [0.027,0.303] | [0.043,0.349] | [-0.201,0.057] | [-0.288,-0.012] | [0.023,0.265] | [-0.014,0.273] |
| Consistently high | 0.917** | 1.124** | 0.680** | 0.828** | -0.432** | -0.655** | 0.487** | 0.652** |
|  | [0.679,1.156] | [0.842,1.405] | [0.443,0.917] | [0.534,1.122] | [-0.664,-0.200] | [-0.900,-0.411] | [0.248,0.725] | [0.389,0.915] |
| Siblings: |  |  |  |  |  |  |  |  |
| Consistently low | Ref. | Ref. | Ref. | Ref. | Ref. | Ref. | Ref. | Ref. |
| Increasing | 0.204** | 0.264** | 0.198** | 0.204** | -0.159 | -0.165* | 0.119 | 0.182** |
|  | [0.069,0.338] | [0.130,0.397] | [0.065,0.331] | [0.064,0.343] | [-0.320,0.001] | [-0.301,-0.030] | [-0.028,0.266] | [0.050,0.314] |
| Decreasing | 0.089 | 0.023 | 0.062 | 0.030 | -0.053 | -0.069 | 0.040 | 0.061 |
|  | [-0.004,0.182] | [-0.093,0.139] | [-0.035,0.160] | [-0.088,0.148] | [-0.150,0.045] | [-0.169,0.031] | [-0.082,0.162] | [-0.045,0.167] |
| Consistently high | 0.298** | 0.300** | 0.252** | 0.285** | -0.139* | -0.237** | 0.191** | 0.234** |
|  | [0.185,0.411] | [0.178,0.422] | [0.137,0.367] | [0.163,0.408] | [-0.260,-0.019] | [-0.351,-0.124] | [0.069,0.314] | [0.120,0.347] |

**Table S7. Sensitivity analysis: Linear regression models showing the association between changes in victimization by peers and siblings and changes in depressive symptoms, life satisfaction, self-esteem and body image between ages 11 and 14, excluding only children (n = 12,802)**

|  | Depressive symptoms a | | Life satisfaction a | | Self esteem b | | Body image a | |
| --- | --- | --- | --- | --- | --- | --- | --- | --- |
|  | Males | Females | Males | Females | Males | Females | Males | Females |
|  | β [99% CI] | β [99% CI] | β [99% CI] | β [99% CI] | β [99% CI] | β [99% CI] | β [99% CI] | β [99% CI] |
| Baseline level of outcome | 0.128** | 0.202** | 0.134** | 0.154** | 0.239** | 0.243** | 0.205** | 0.218** |
|  | [0.089,0.167] | [0.151,0.252] | [0.094,0.174] | [0.109,0.200] | [0.199,0.279] | [0.201,0.286] | [0.160,0.250] | [0.176,0.259] |
| Peers: |  |  |  |  |  |  |  |  |
| Consistently low | Ref. | Ref. | Ref. | Ref. | Ref. | Ref. | Ref. | Ref. |
| Increasing | 0.578** | 0.946** | 0.431** | 0.731** | -0.309** | -0.624** | 0.351** | 0.572** |
|  | [0.431,0.725] | [0.753,1.138] | [0.282,0.580] | [0.545,0.918] | [-0.489,-0.129] | [-0.788,-0.459] | [0.183,0.519] | [0.405,0.740] |
| Decreasing | 0.036 | 0.080 | 0.120 | 0.130 | 0.002 | -0.037 | 0.086 | 0.029 |
|  | [-0.090,0.162] | [-0.071,0.230] | [-0.018,0.257] | [-0.024,0.285] | [-0.119,0.124] | [-0.170,0.097] | [-0.033,0.206] | [-0.112,0.170] |
| Consistently high | 0.794** | 0.872** | 0.609** | 0.737** | -0.297** | -0.485** | 0.385** | 0.505** |
|  | [0.566,1.023] | [0.590,1.154] | [0.372,0.847] | [0.459,1.016] | [-0.512,-0.082] | [-0.728,-0.243] | [0.155,0.615] | [0.245,0.766] |
| Siblings: |  |  |  |  |  |  |  |  |
| Consistently low | Ref. | Ref. | Ref. | Ref. | Ref. | Ref. | Ref. | Ref. |
| Increasing | 0.197** | 0.262** | 0.194** | 0.201** | -0.150 | -0.169* | 0.115 | 0.176** |
|  | [0.065,0.329] | [0.128,0.396] | [0.063,0.324] | [0.064,0.338] | [-0.308,0.008] | [-0.305,-0.034] | [-0.027,0.257] | [0.046,0.306] |
| Decreasing | 0.061 | -0.042 | 0.037 | 0.002 | -0.008 | -0.021 | 0.002 | 0.023 |
|  | [-0.031,0.153] | [-0.158,0.074] | [-0.061,0.135] | [-0.111,0.116] | [-0.104,0.088] | [-0.117,0.076] | [-0.118,0.122] | [-0.079,0.125] |
| Consistently high | 0.258** | 0.238** | 0.227** | 0.255** | -0.110 | -0.195** | 0.161** | 0.188** |
|  | [0.145,0.370] | [0.119,0.358] | [0.115,0.339] | [0.133,0.377] | [-0.225,0.005] | [-0.307,-0.083] | [0.042,0.279] | [0.075,0.301] |

Note. * p < 0.01, ** p < 0.001. ^a^ positive coefficients indicate greater deterioration in outcomes, ^b^ positive coefficients indicate greater improvement outcomes. All models are adjusted for age, sex, ethnicity, family income, living in a two-parent household, being an only child, age 8 emotional problems, special educational needs, long term illness, age 11 BMI percentile, BMI percentile change between age 11 and age 14, and pubertal development.
